# Porcine submandibular glands as potential salivary gland experimental models: histological, immunohistochemical, and ultrastructural characterization

**DOI:** 10.1007/s10735-026-10898-w

**Published:** 2026-07-14

**Authors:** Helena Ab’Sáber Simões, Cibele Pelissari, Giovanna Florezi, Ricardo Hsieh, Victor Elias Arana-Chavez, Cristina Massoco, Christine Delporte, Alexandre Ab’Saber, Silvia Vanessa Lourenço

**Affiliations:** 1 Department of Surgery, Faculdade de Medicina, Universidade de São Paulo, Av. Dr. Arnaldo, 455 - São Paulo, São Paulo, SP - 01246-903 Brazil; 2 LIM-06, Faculdade de Medicina, Instituto de Medicina Tropical, Universidade de São Paulo, São Paulo, Brazil; 3 Laboratory of Pathophysiological and Nutritional Biochemistry, Medical School, Université Libre de Bruxelles, Brussels, Belgium; 4 Department of Biomaterials and Oral Biology, Faculdade de Odontologia, Universidade de São Paulo, São Paulo, Brazil; 5 Department of Pathology and Toxicology, Faculdade de Medicina Veterinária e Zootecnia, Universidade de São Paulo, São Paulo, Brazil; 6 Division of Anatomical Pathology, Hospital das Clínicas, Faculdade de Medicina da Universidade de São Paulo, São Paulo, Brazil

**Keywords:** Salivary glands, Swine, Translational medical research, Immunophenotyping, Microscopy, Electron, Transmission

## Abstract

Salivary glands play an essential role in oral homeostasis by producing saliva, which protects oral tissues and maintains the oral environment. Despite growing interest in porcine models for translational biomedical research, the immunophenotypic characterization of porcine salivary glands remains limited in the literature, with few studies addressing their cytokeratin and contractile protein expression profiles. This gap constrains the ability to directly compare porcine and human glandular phenotypes and hinders the establishment of the pig as a validated salivary gland experimental model. This study evaluates the histological, immunophenotypic, and ultrastructural features of porcine submandibular glands as potential experimental models. Submandibular glands from 11 pigs aged 3 to 6 months (average weight: 20 kg) were examined histologically using hematoxylin and eosin staining and ultrastructurally by transmission electron microscopy, and phenotypically via immunohistochemistry for key markers such as CK5, CK7, CK19, SMA, calponin, caldesmon and S-100. Porcine submandibular glands exhibit a lobular organization akin to human glands, with mucous acinar cells, intercalated and excretory ducts, and rich vascularization. Immunohistochemistry revealed cytokeratins in epithelial cells and contractile proteins in myoepithelial cells, mirroring human glandular markers. Ultrastructural analysis highlighted robust cellular junctions, myoepithelial support, and intricate nerve fiber networks essential for glandular function. The structural and phenotypic parallels between porcine and human submandibular salivary glands provide a descriptive basis supporting their potential use in comparative salivary gland research.

## Introduction

Salivary glands are exocrine organs responsible for the secretion of saliva, which is essential for maintaining homeostasis in the oral cavity. This fluid, composed primarily of water along with proteins and inorganic components, performs multiple functions including lubrication of mucosal surfaces, protection of oral structures, and maintenance of the oral microbiome. Saliva is synthesized in secretory end pieces, composed of secretory cells surrounded by myoepithelial cells and is transported by an intricate ductal system, which modulates saliva composition (de Paula et al. 2017). The autonomic nervous system controls saliva secretion, which is based on cholinergic signaling by parasympathetic nerves and adrenergic signaling by sympathetic nerves. Recent studies describe an increasing number of people that experience xerostomia due to ageing, menopause, inflammatory and autoimmune disease, metabolic disease, radiotherapy, and other systemic conditions; they have also established an association between salivary function and quality of life (de Paula et al. 2017; Togni et al. 2019; Chibly et al. 2022).

Studies on the structure and physiology of salivary glands have been mainly performed in small rodents with important advances in the understanding of glandular biology. Despite rodent glands being used as the main experimental models in salivary gland research, they bear limited similarities with their human counterparts; peculiarities, including development, short lifespan, eating habits and other biological factors hamper direct comparisons of salivary gland physiology and physiopathology and are challenges for researchers seeking to investigate disease mechanisms using these animals (Wang et al. 2007; Zhang et al. 2018). Furthermore, while the human submandibular gland is a mixed gland containing both serous and mucous acini, the submandibular gland of rodents is exclusively serous, a fundamental histological difference that limits the translational relevance of rodent models for studies involving mucus secretion and glandular composition (Amano et al. 2012). In addition, the submandibular salivary gland is the most extensively studied, as it is the largest salivary gland in rodents. However, it contains the granular convoluted tubule, a segment of the duct system located between the intercalated and striated ducts, which is not present in humans. The cells of the granular convoluted tubule synthesize a wide variety of biologically active polypeptides that exert specific functions in rodent metabolism. Indeed, this well-developed portion of the duct system accounts for 47–65% of the gland in males and 19–36% in females (Gresik 1994).

Porcine models are considered genetically similar to humans, with significant biological compatibility (Joshi et al. 2024). Their organs have been used for comparative functional studies and for medical therapy, such as in heart and kidney xenotransplantation (Yang and Sykes 2007). Beyond systemic organs, pigs share several craniofacial anatomical structures with humans, including the mandible, maxilla, and oral mucosal organization, and their salivary glands are positioned and structured in a manner broadly comparable to human major salivary glands (Štembírek et al. 2012).

Porcine salivary glands have been neglected as models for comparative studies with humans due to animal costs and handling difficulties. However, at present, porcine salivary glands may be a reliable alternative for new advances in research aiming regenerative approaches (Zhou et al. 2010; Schachtschneider et al. 2017). A few published studies have compared the structural similarities of human and porcine salivary glands. Early histological and ultrastructural descriptions of the porcine parotid gland established the distinctive features of porcine secretory cells, including serous acini with electron-lucent secretory granules and mitochondria with tubular cristae (Boshell and Wilborn 1978). Subsequent studies characterized the submandibular gland of miniature pigs anatomically, histologically, histochemically, and ultrastructurally, confirming its structural comparability with human glands and establishing the pig as a candidate model for biomedical research (Zhang et al. 2005). The porcine model has also been applied in the study of radiation-induced salivary gland injury, demonstrating structural and functional responses comparable to those described in human patients (Radfar and Sirois 2003). More recently, Zhang et al. (2018) examined tight junction protein expression across porcine, human, and murine glands, but did not analyze the broader phenotypic profile of these tissues, which is essential for comparative research (Zhang et al. 2018).

The submandibular gland was selected for this study because it is one of the major salivary glands responsible for basal salivary secretion, has been extensively investigated in studies of salivary gland physiology and regenerative medicine, and presents easier surgical access and tissue sampling compared to other salivary glands.

This study aimed to characterize the histological, ultrastructural, and immunophenotypic features of porcine submandibular glands and to compare these characteristics with those reported for human salivary glands.

## Methods

### Histological and ultrastructural analysis

Submandibular salivary glands were collected from 11 domestic pigs *(Sus scrofa domesticus)*, Landrace x Large White crossbred, aged 3 to 6 months, with an average body weight of 20 kg, operated in the surgical training theatre of the Medical School, University of São Paulo. The number of specimens included in the study was determined according to tissue availability and ethical principles aimed at minimizing animal use. All procedures involving animals were conducted according to ARRIVE 2.0 guidelines (Percie Du Sert et al. 2020) and approved by the local ethical committee (protocol #003/2023-I). The specimens were prepared for histological and ultrastructural analysis.

For the histological analysis, the specimens were fixed in buffered formalin, histologically processed and the resulting slides were stained with conventional haematoxylin and eosin.

Specimens for transmission electron microscopy (TEM) analysis were prepared following standard protocols to ensure high-quality ultrathin sections. Initially, the samples were fixed in 2.5% glutaraldehyde in 0.1 M phosphate buffer (pH 7.4) for 2 h at 4 °C to preserve cellular structures. Post-fixation was carried out using 1% osmium tetroxide (OsO₄) for 1 h at room temperature, enhancing contrast by staining lipid membranes.

After fixation, the samples were dehydrated in a graded ethanol series (30%, 50%, 70%, 90%, and 100%) and subsequently embedded in epoxy resin. Ultrathin Sects.  (60–70 nm) were obtained using an ultramicrotome equipped with a diamond knife and placed on 200-mesh copper and nickel grids coated with Parlodium (Electron Microscopy Sciences, Hatfield, PA, USA). To enhance contrast, ultrathin sections were stained with 2% uranyl acetate for 10 min followed by lead citrate for 5 min. These staining steps improve electron scattering, providing better visualization of cellular and subcellular structures. Finally, the material was examined with a 1010 JEOL transmission electron microscope (JEOL Ltd., Akishima, Tokyo, Japan).

### Immunohistochemical phenotyping

Serial sections (4 μm) of porcine submandibular salivary glands were deparaffinised, re-hydrated and submitted to antigen retrieval. The sections were incubated in 3% aqueous hydrogen peroxide for 15 min to quench endogenous peroxidase activity, followed by Protein Block Serum-Free incubation (DakoCytomation, Carpinteria, CA, USA) for 20 min at room temperature to suppress nonspecific binding. Primary antibodies were incubated overnight at 4 °C, followed by incubation with the indirect dextran polymer detection system (En Vision—Dako, Carpinteria, CA, USA). Information of primary antibodies and protocols employed are described in Table 1. The reactions were revealed with incubation with 3′3 diaminobenzidine tetrachloride (DAB) for 3 min at room temperature and counterstained with Carazzi’s haematoxylin. Negative controls were incubated only with non-immune serum instead of the primary antibody, and positive controls were considered according to the manufacturer’s datasheet recommendation.

The specimens were analyzed by two experienced pathologists. A qualitative evaluation of each protein expression was performed using a conventional optical microscope (Olympus E330).

**Table 1:** – Primary antibodies source, dilution and antigen retrieval method

Primary antibody	Research resource identifiers (RRID)	Dilution	Antigen retrieval	Positive controls
CK5	Sigma-Aldrich Cat# C5992, RRID: AB_2134432	1:100	Citrate buffer pH 6,0	Human Skin
CK7	Cell Marque Cat# 307 M-95, RRID: AB_1159482	1:100	Citrate buffer pH 6,0	Human Placenta
CK19	Cell Marque Cat# 319R-14, RRID: AB_1158239	1:100	Citrate buffer pH 6,0	Human Liver
SMA	Cell Marque Cat# 202 M-94, RRID: AB_1157937	1:100	Citrate buffer pH 6,0	Human Colon
S-100	Agilent Cat# IS504, RRID: AB_3099743	Ready to use	Citrate buffer pH 6,0	Human Skin
Caldesmon	Abcam Cat# ab68878, RRID: AB_1924845	1:200	Citrate buffer pH 6,0	Human Placenta
Calponin	Abcam Cat# ab46794, RRID: AB_2291941	1:300	Citrate buffer pH 6,0	Human Breast

## Results

### Histological analysis

**Fig. 1:**
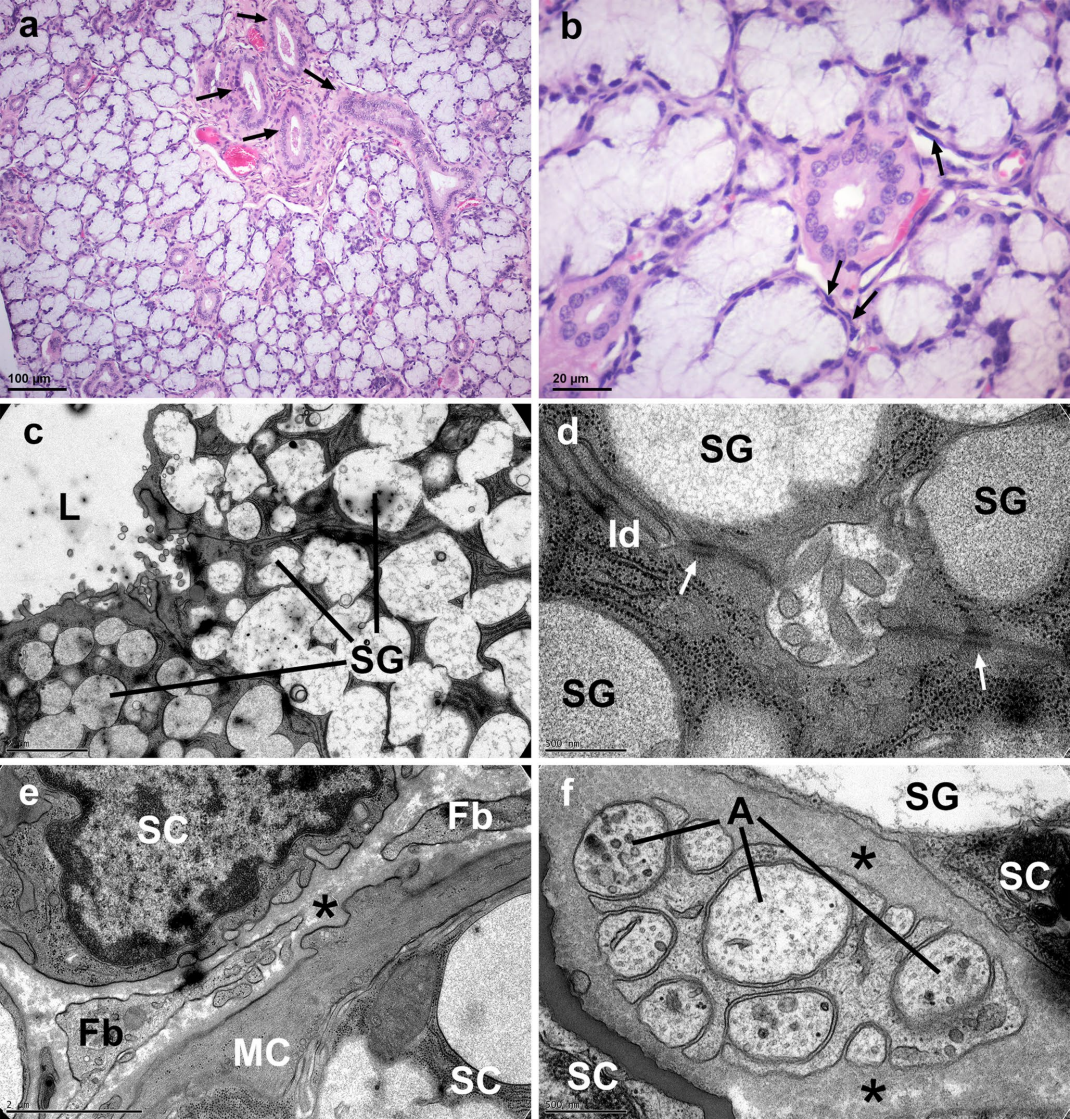
Histological and ultrastructural analysis of the porcine submandibular salivary gland specimens. **a** Porcine submandibular salivary gland showing predominantly mucous secretory end pieces. Note an interlobular excretory duct with ramifications across the glandular parenchyma and its proximity to congested blood capillaries (arrows) (HE, 100×); **b** Intralobular excretory duct and its close proximity to blood capillaries. Note the myoepithelial cells (arrows) surrounding the mucous secretory end pieces (HE, 400×); **c** Numerous secretory granules (SG) are visible at the apical pole of secretory cells, near the lumen (L) of the secretory end piece; **d** Region between two adjacent secretory cells, characterized by interdigitations (Id) and desmosomes (arrows). Secretory granules (SG) are also visible within the secretory cells; **e** Portion of a myoepithelial cell (MC) is seen surrounding a secretory cell (SC). In the adjacent connective septum (*) portions of fibroblasts (Fb) are present; **f** An unmyelinated nerve fiber showing several axons (A) in cross-section, located in the connective tissue (*) that fills the septum between the secretory end pieces. Two small portions of secretory cells (SC) and a secretory granule (SG) are also visible.

The submandibular salivary glands from the 11 pigs included in the study were composed of multiple lobules of mucous acinar cells. The secretory cells were large and pyramidal, with clear cytoplasm filled with a clear content, interpreted as mucus; they were connected to intercalated ducts, which consisted of a single layer of ductal cells and myoepithelial cells (Fig. 1a). Fusiform-shaped myoepithelial cells surrounded the secretory end pieces. Intralobular ducts with eosinophilic epithelial lining suggestive of striated ducts were observed, interwoven by a complex network of blood microvessels (Fig. 1b). The multiple acinar lobules were interconnected by larger extralobular excretory ducts, composed of eosinophilic luminal cells and basal cells, located near blood vessels.

### Ultrastructural analysis

Ultrastructural examination showed secretory cells containing numerous secretory granules with fine material especially at their apical portion, near the lumen of the secretory end pieces (Fig. 1c). Numerous interdigitations between the cells were observed at their basolateral plasma membranes, in addition to desmosomes and other intercellular junctions (Fig. 1d). These structures revealed a complex architecture and the essential role they play in ensuring strong intercellular adhesion for maintaining the gland’s structural integrity.

At higher magnifications, fusiform shaped myoepithelial cells were identified surrounding the secretory end pieces, conspicuously arranged inside the basal lamina in close relationship with the secretory cells (Fig. 1e).

The ultrastructure analysis of the connective tissue that is located between the secretory end pieces showed fusiform fibroblasts surrounded by an extracellular matrix with collagen fibrils among which blood vessels and nerve fibers were present. Typical unmyelinated fibers were observed into the connective tissue, thus representing an epilemmal relationship with the secretory end pieces (Fig. 1f).

### Immunophenotyping

**Fig. 2:**
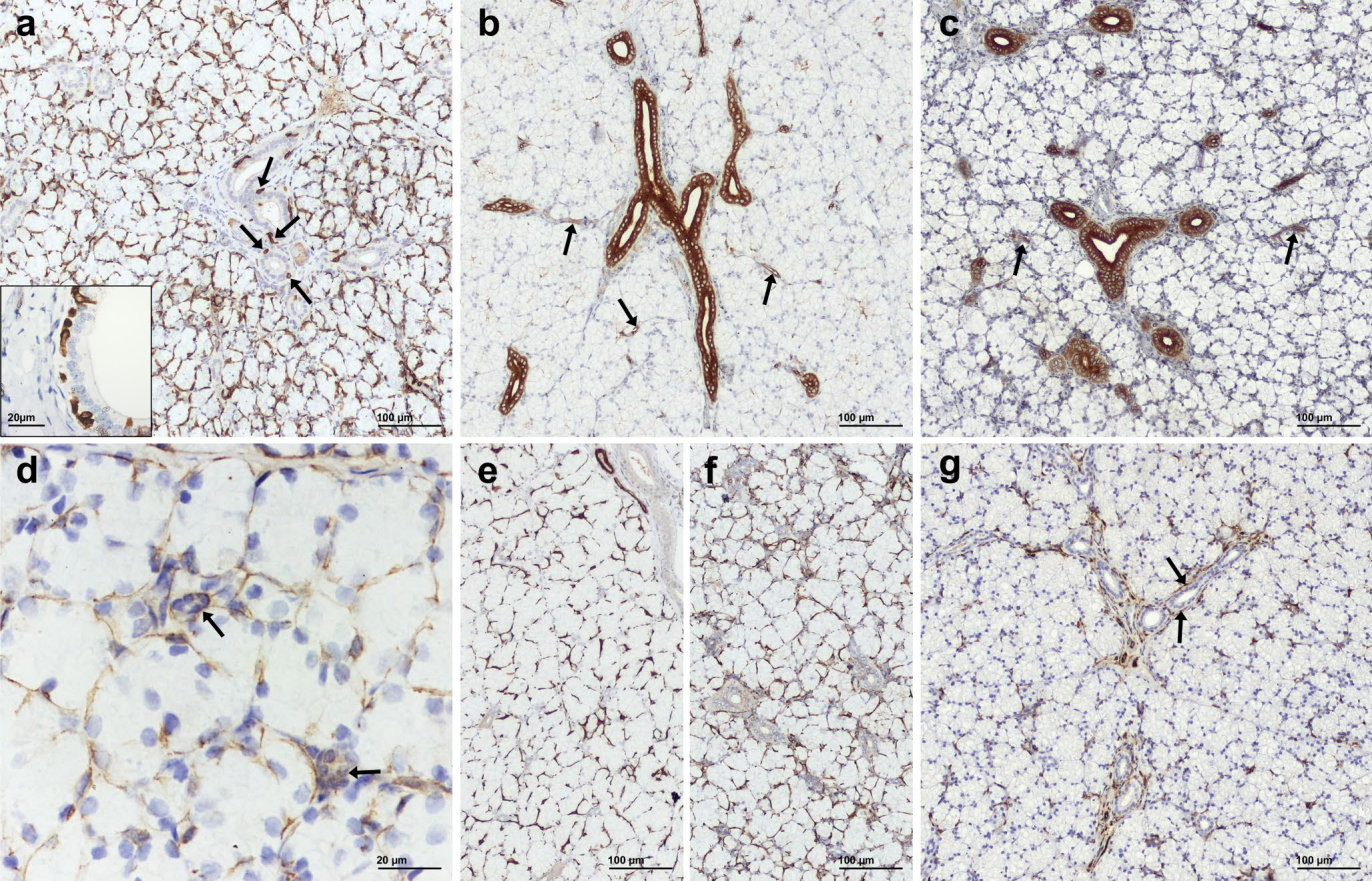
Porcine submandibular salivary gland specimens immunophenotyping. **a**: Ductal basal cells and myoepithelial cells positive for CK5. In some duct structures, scattered CK5-positive basal cells surround the luminal cell layer (arrows). Inset: higher magnification showing CK5-positive basal cells in an intralobular excretory duct (×400). Original magnification, ×100; **b**: Luminal cells of the intralobular duct ramifications positive for CK7. Luminal cells of intercalated ducts are also CK7-positive (arrows). Original magnification, ×100; **c**: CK19 expression in luminal cells of intralobular excretory ducts and intercalated ducts (arrows). Original magnification, ×100; **d-f**: Expression of contractile proteins in myoepithelial cells surrounding mucous secretory end pieces and intercalated ducts: smooth muscle actin (d, ×400), calponin (e, ×100), and caldesmon (f, ×100). Arrow in (d) indicates myoepithelial cells around an intercalated duct; **g**: S-100 expression in myoepithelial cells and nerve fibers adjacent to excretory ducts (arrows). Original magnification, ×100.

The immunohistochemical analysis of porcine submandibular salivary glands revealed CK5 positivity in the cytoplasm of basal and myoepithelial cells (Fig. 2a). Strong CK7 staining was observed in ductal epithelial cells (Fig. 2b). Cytokeratin 19 (CK19) was expressed in excretory ducts and myoepithelial cells, as depicted in Fig. 2c. Additionally, myoepithelial cells expressed a set of contractile proteins - smooth muscle actin (SMA), (Fig. 2d), calponin (Fig. 2e), caldesmon (Fig. 2f) as well as S-100 (Fig. 2g).

## Discussion

The present study provides a systematic immunophenotypic characterization of the porcine submandibular gland using antibodies validated for human tissue and demonstrates that its structural and phenotypic organization is broadly comparable to that of the human gland. The lobular architecture, hierarchical ductal system, contractile protein expression of myoepithelial cells, and epilemmal innervation pattern align with classical descriptions of mammalian salivary gland morphology (Garrett 1987; Tandler 1993 a, b; Tandler and Phillips 1993; Vanelli et al. 2026).

Histologically, the porcine submandibular salivary glands exhibited the typical lobular organization of an exocrine gland, composed predominantly of pyramidal mucous acinar cells with clear cytoplasm reflecting abundant mucin content. Although both porcine and human submandibular glands exhibit mixed secretory organization, the human submandibular gland is classically described as predominantly serous, whereas the porcine glands analyzed exhibited predominance of mucous secretory units, in agreement with previous descriptions of the porcine submandibular gland (de Paula et al. 2017).

The predominance of mucous secretory units observed in these specimens should be interpreted in light of the developmental stage of the animals. In pigs, the composition of the submandibular gland undergoes androgen-dependent remodeling after puberty, resulting in marked sexual dimorphism: mature boars develop predominantly serous secretory units with serous cell hypertrophy, whereas females and castrated males retain a predominance of mucous secretory units. However, this sexually dimorphic pattern is considered an androgen-dependent post-pubertal phenomenon. The animals included were 3 to 6 months of age, corresponding to the developmental period that precedes puberty and the first estrous cycle in this species, a developmental window during which the proportion of serous and mucous cells does not differ significantly between sexes (Booth et al. 1973). The predominance of mucous secretory units observed in our specimens aligns with the pre-pubertal glandular composition described for pigs of both sexes at this age, before the onset of androgen-dependent sexual dimorphism. These findings indicate that the suitability of the porcine submandibular gland as an experimental model depends on the developmental stage of the animals used. Consequently, studies specifically addressing serous secretory physiology should carefully consider both developmental stage and sex when selecting this model. This developmental pattern is consistent with previous histological descriptions of mammalian salivary glands, including those of humans (de Paula et al. 2017).

Intercalated ducts, formed by a single layer of ductal cells and surrounded by myoepithelial cells, highlight their role in the efficient transport and modification of saliva, agreeing with previous reports describing intercalated ducts as key regulators of salivary composition (Porcheri and Mitsiadis 2019). The hierarchical organization of the ductal system in our sample, with a clear progression from intercalated to intralobular and extralobular ducts, parallels that described during human salivary gland development, in which ductal lumen formation and epithelial differentiation follow a coordinated morphogenetic programme (Teshima et al. 2016).

Fusiform myoepithelial cells were detected around the secretory end pieces, highlighting their role in providing structural support and facilitating glandular secretion through their contractile properties (Proctor 2016).

The principal histological similarities and differences between porcine and human salivary glands reported in the literature are summarized in Table 2. Porcine submandibular glands closely mirror the structural organization described in human salivary glands, indicating their use as a comparative model for morphological and translational studies.

Ultrastructural examination demonstrated well-preserved secretory cells, myoepithelial cells, connective tissue components, and neural elements, providing further insight into the structural organization of the porcine submandibular gland. The close association between myoepithelial and secretory cells, together with the presence of cytoplasmic filament bundles, intimate association with the basal lamina, and cytoplasmic processes embracing the secretory end pieces, mirrors the classical ultrastructural description of mammalian salivary gland myoepithelial cells (Redman 1994). These cytoplasmic extensions may facilitate and amplify the contraction potential of secretory units, aiding expulsion of saliva into the oral cavity (Chitturi et al. 2015).

Electron microscopy analysis also demonstrated nerve fibers and their subcellular components, such as axons and their vesicles, suggesting the fundamental role of these structures in neural communication and in the regulation of the gland’s secretory functions (Proctor 2016; D’Alessandro and Meldolesi 2019). The epilemmal distribution of unmyelinated nerve fibers observed in the connective tissue septa between secretory end pieces matches the established patterns of salivary gland autonomic innervation, in which parasympathetic and sympathetic fibers regulate secretion through both vascular and direct parenchymal pathways (Garrett 1987; Garrett and Kidd 1993).

The ultrastructural findings, including cell interdigitations, desmosomes, and the intimate relationship between secretory cells, myoepithelial cells, and connective tissue, are in line with classic descriptions of mammalian salivary gland ultrastructure (Tandler 1993 a, b; Tandler and Phillips 1993). These intercellular junctions contribute to cell adhesion and help maintain glandular structural integrity during the dynamic processes of secretion and contraction (Samiei et al. 2019).

Immunohistochemical analysis further confirmed the myoepithelial phenotype through the expression of SMA, calponin, caldesmon and S-100. These proteins are well-established markers of myoepithelial differentiation in human salivary glands and other exocrine tissues, and their expression in the porcine submandibular gland suggests that the immunophenotypic profile of these cells is highly conserved across species, reinforcing the suitability of the porcine gland as a comparative experimental model (Campbell et al. 1988; Donato 1991; Fanò et al. 1995; Chitturi et al. 2015). Previous studies have shown that SMA and other contractile proteins participate in branching morphogenesis during human salivary gland development (Ianez et al. 2010), however, comprehensive characterization of these markers in porcine salivary glands has remained limited.

Maintenance of salivary gland homeostasis relies heavily on the contractile activity of myoepithelial cells by providing mechanical support to the secretory end pieces and facilitating saliva secretion (Amano et al. 2025). The coordinated expression of SMA, calponin, and caldesmon reinforces the presence of the contractile machinery required for these functions. SMA identifies the contractile cytoskeleton of myoepithelial cells, whereas calponin, a calcium- and actin-binding protein, regulates actomyosin interactions during contraction (Jensen et al. 2014; Hsieh and Jin 2023). Caldesmon complements this process by modulating actin–myosin interactions while stabilizing actin filaments, thereby contributing to both cytoskeletal organization and coordinated contraction (Goncharova et al. 2001; Hai and Gu 2006). Combined, the expression of SMA, calponin, and caldesmon indicates the presence of a well-developed contractile apparatus in porcine myoepithelial cells, closely resembling the functional phenotype described in human salivary glands (Gimona et al. 2003).

In addition to their expression in myoepithelial cells, SMA, calponin and caldesmon were also detected in the rich network of blood vessels and intertwined with the intralobular ducts, which emphasizes the importance of adequate vascularization for cellular metabolism and the maintenance of glandular homeostasis; these findings were similar to data described in comparative histological studies of salivary glands performed by other investigators (Aure et al. 2015; de Paula et al. 2017). Although functional parameters were not evaluated, previous investigations have demonstrated that salivary flow rate, pH, and ionic composition (Na⁺, K⁺, Ca²⁺) in miniature pigs more closely resemble those of humans than those of rats, reinforcing the value of porcine models for studies of salivary gland function and composition (Li et al. 2007). Together with the structural, ultrastructural, and immunophenotypic findings, these functional similarities further indicate the value of the porcine submandibular gland as a translational experimental model.

Beyond these morphological similarities, the cytokeratin expression pattern further demonstrated conservation of epithelial compartmentalization between porcine and human salivary glands (Fuchs and Raghavan 2002; Wang et al. 2007; Zhou et al. 2010). CK5 expression was restricted to basal and myoepithelial cells, mirroring its recognized role as a marker of epithelial progenitor populations and tissue maintenance in several exocrine organs (Fuchs and Raghavan 2002; Raimondi et al. 2006; Moll et al. 2008; Alam et al. 2011).

CK7 showed strong expression throughout the ductal epithelium, confirming its association with secretory epithelia (Azevedo et al. 2008; Rao et al. 2014). Cytokeratin 19 was also detected in porcine submandibular glands, representing a novel contribution to the immunophenotypic characterization of porcine salivary tissue, mirroring its distribution in human salivary glands, where it contributes to epithelial organization and ductal integrity supporting glandular function (Zhang et al. 2024).

Taken together, the cytokeratin profile reproduced the epithelial compartmentalization described in human salivary glands, with CK5 identifying the basal cell population and CK7/CK19 predominating in the ductal epithelium. Combined with the contractile marker profile, these findings demonstrate that porcine submandibular glands share not only a similar histological organization but also a conserved epithelial and myoepithelial immunophenotype, further reinforcing their use as a translational experimental model.

Major strengths of this study include the combined histological, ultrastructural and immunophenotypic characterization of porcine submandibular glands using antibodies routinely applied to human tissue. This study has some limitations. First, no human salivary gland specimens were analyzed in parallel, all comparisons with human tissue rely on published literature, which limits the strength of the translational conclusions. In addition, the analyses performed were qualitative and descriptive in nature. Although available evidence indicates minimal sexual dimorphism before puberty, the sex of individual animals was not recorded and therefore a residual influence of sex cannot be formally excluded. Future studies should include balanced male and female cohorts to better characterize possible sex-related differences and their implications for translational applications. Also, the sample size was determined by tissue availability and ethical minimization principles, in the absence of a formal power calculation. Although all antibodies produced staining patterns consistent with the expected cellular distribution, they were originally validated for human tissues, and species-specific differences in antigen expression or antibody affinity cannot be completely excluded. Future studies including quantitative analyses, human controls, and functional assays are necessary to further validate the porcine submandibular gland as a translational model.

Future investigations evaluating extracellular matrix components and additional salivary gland biomarkers may contribute to a more comprehensive characterization of porcine salivary glands and their potential use in comparative and regenerative studies.

The morphological and phenotypic evidence gathered here provides a comprehensive framework for future comparative studies. Further validation using human tissue processed in parallel, together with quantitative and functional analyses, will be essential to establish the porcine submandibular gland as a reliable experimental model for studies of regeneration, radiation-induced injury, xerostomia, and other disorders affecting salivary gland function.

**Table 2:** – Porcine and human salivary glands comparative analysis in the literature

Glandular structures		Human salivary glands	Porcine salivary glands	References
Secretory end pieces	Secretory cells	Large cells with clusters of pyramidal or rounded cells organized into serous (eosinophilic granules, spherical nuclei), mucous (clear cytoplasm, basal nuclei), or mixed units. No sexual dimorphism.	Pyramidal or cuboidal cells arranged in secretory units. Parotid is predominantly pure serous; submandibular/sublingual are mixed. Morphologically and functionally similar to humans, requiring dual autonomic stimulation for secretion. Boars exhibit significant serous cell hypertrophy compared to females.	Tandler 1993 a; Tandler and Phillips 1993; Zhang et al. 2005, 2018; Zhou et al. 2010; de Paula et al. 2017; Chibly et al. 2022; Rao et al. 2026
	Myoepithelial cells	Stellate or spindle-shaped cells between basal lamina and acinar/ductal cells; contain myofibrils (actin/myosin) for contractile support.	Long, spindle-shaped or stellate epithelial cells with finger-like protrusions. Distributed around acini and intercalated ducts. Structurally and functionally comparable to human counterparts.	Ogawa 2003; Tucker 2007; Mattingly et al. 2015; de Paula et al. 2017
Ductal system	Intercalated ducts	Smallest ductal segments lined by a single layer of simple low cuboidal epithelium with central nuclei. Connect acini to striated ducts and involve initial saliva transport.	Lined by simple cuboidal epithelium; polygonal cells with large indented nuclei. Anatomically and morphologically similar to human ducts.	Tandler 1993 b; de Paula et al. 2017; Chibly et al. 2022; Rao et al. 2026
	Intralobular ducts	Primarily comprised of striated ducts lined by tall columnar epithelium with prominent basal mitochondrial striations for electrolyte modification.	Well-developed striated ducts with prominent acidophilic columnar cells and basal invaginations for ion transport; similar to human architecture	
	Extralobular ducts	Interlobular ducts lined by pseudostratified or stratified columnar epithelium; transitions to stratified squamous near the oral opening	Large ducts located in connective tissue septa; pseudostratified or stratified epithelium; transitions to squamous near oral cavity	

## Data Availability

No datasets were generated or analysed during the current study.
